# Supplementary figures and images for: Comprehensive study of serine/arginine-rich (SR) gene family in rice: characterization, evolution and expression analysis
Source: PeerJ. 2023 Oct 13;11:e16193. doi: 10.7717/peerj.16193 (PMC10578304; doi:10.7717/peerj.16193)

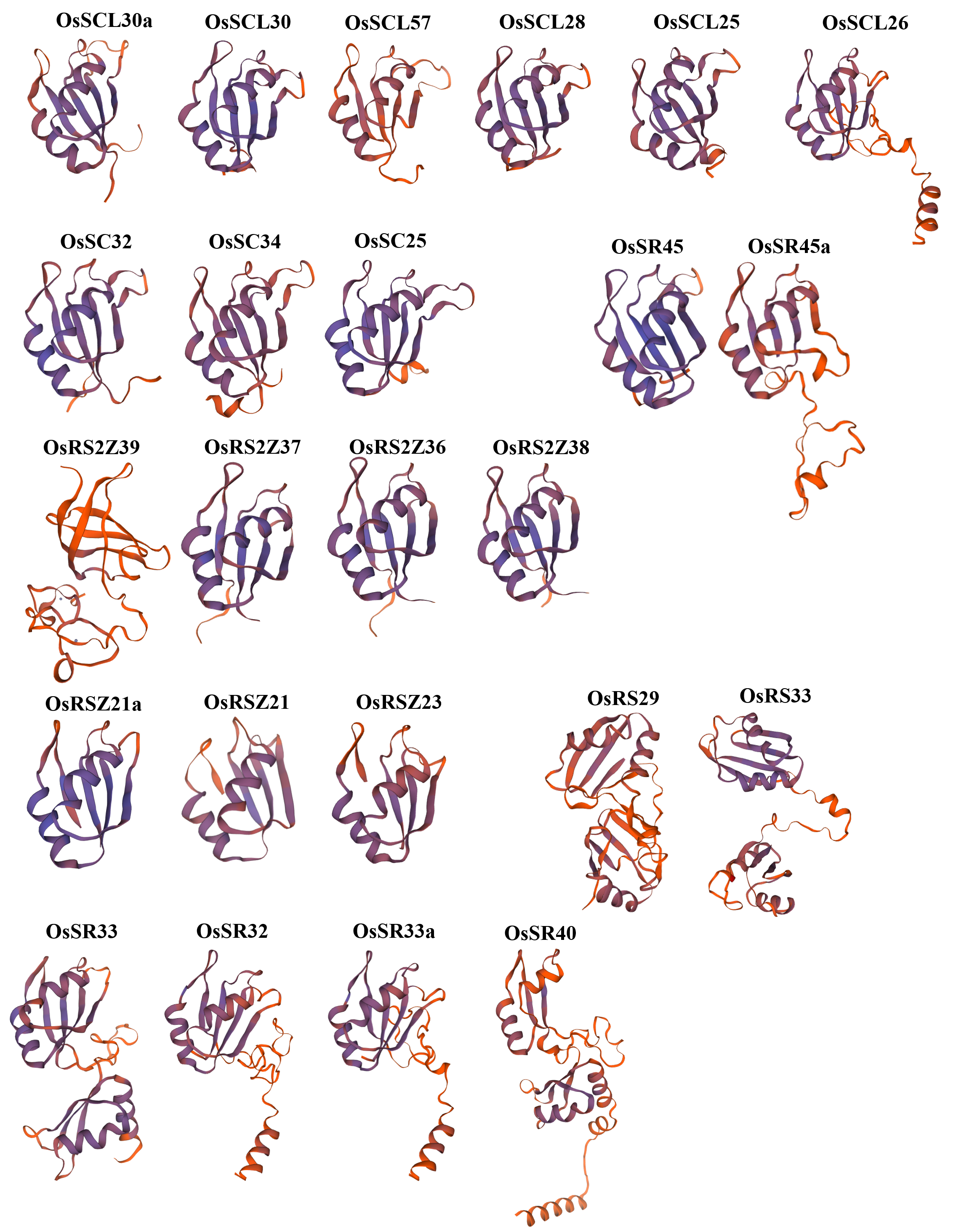

Supplement: Supplemental Information 10 — Predicted three-dimensional domains of OsSR proteins. 3D models of the 24 OsSR proteins according to SWISS-MODEL. [file peerj-11-16193-s010.png]

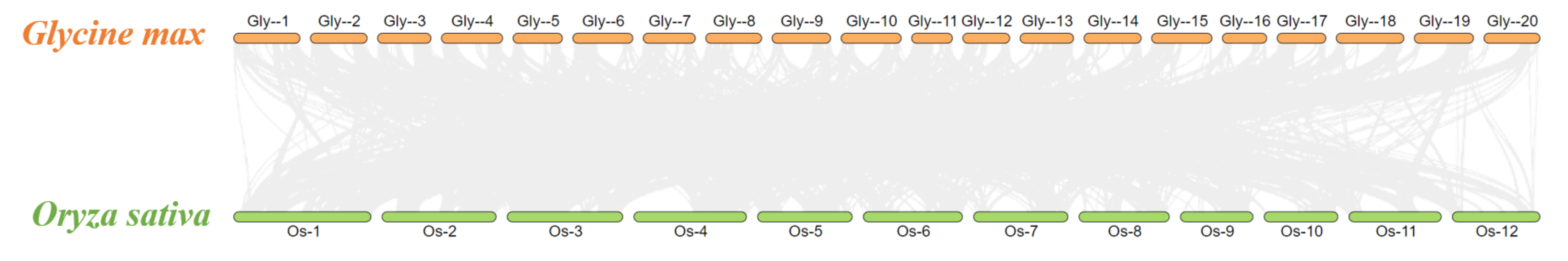

Supplement: Supplemental Information 11 — Gray lines indicated collinear blocks and syntenic SR gene pairs would be highlighted in blue lines. [file peerj-11-16193-s011.png]

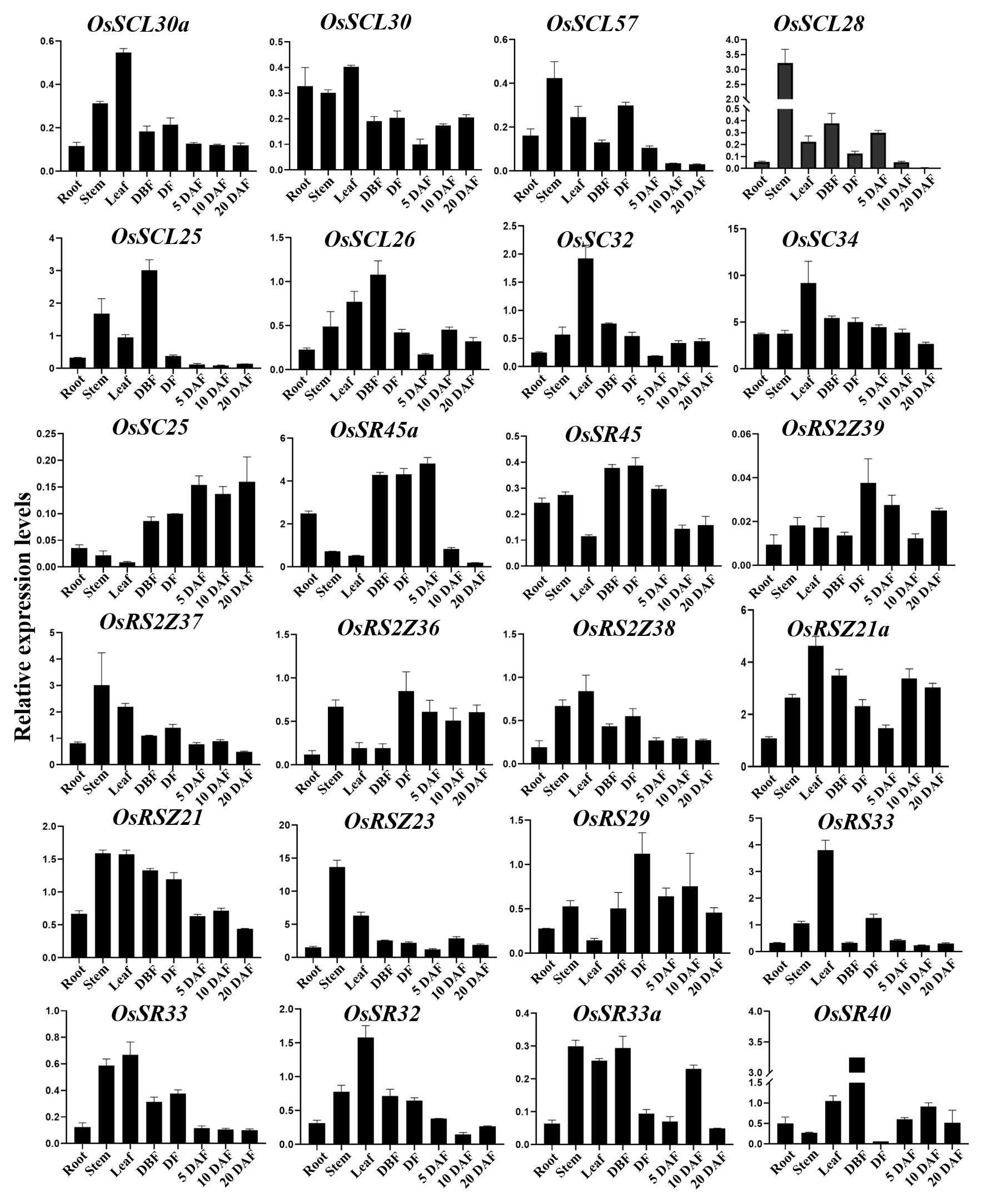

Supplement: Supplemental Information 12 — DBF, Day before fertilization; DF, Day of flowering; DAF, Day after fertilization. OsActin was used as control, error bars represent mean ± SE of three biological replicates. [file peerj-11-16193-s012.png]

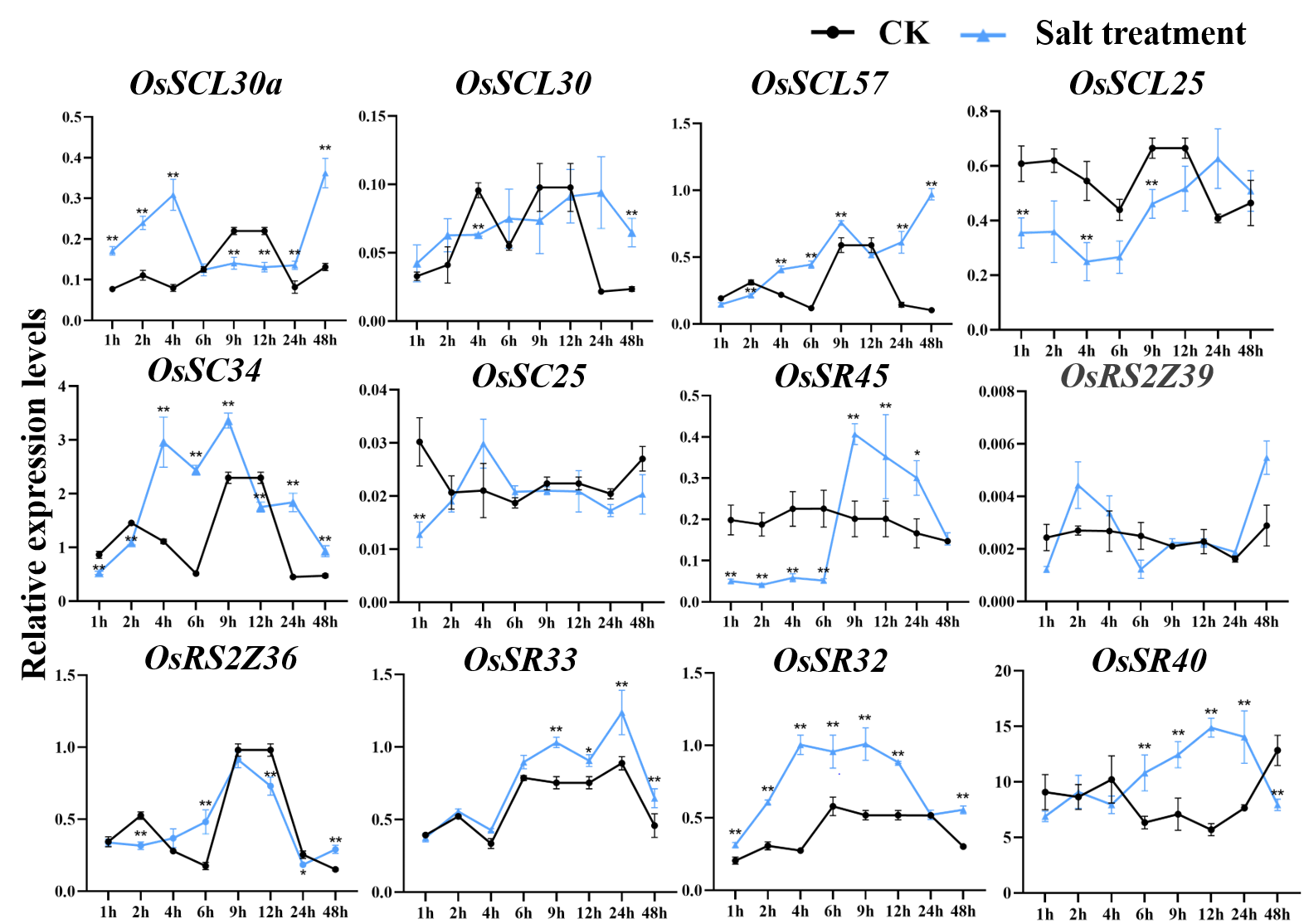

Supplement: Supplemental Information 13 — OsActin was used as control. Error bars represent mean ±SE of three biological replicates. ∗P < 0.05 and ∗∗P < 0.01 indicate significant differences com-pared with CK determined by Student’s t-test. [file peerj-11-16193-s013.png]

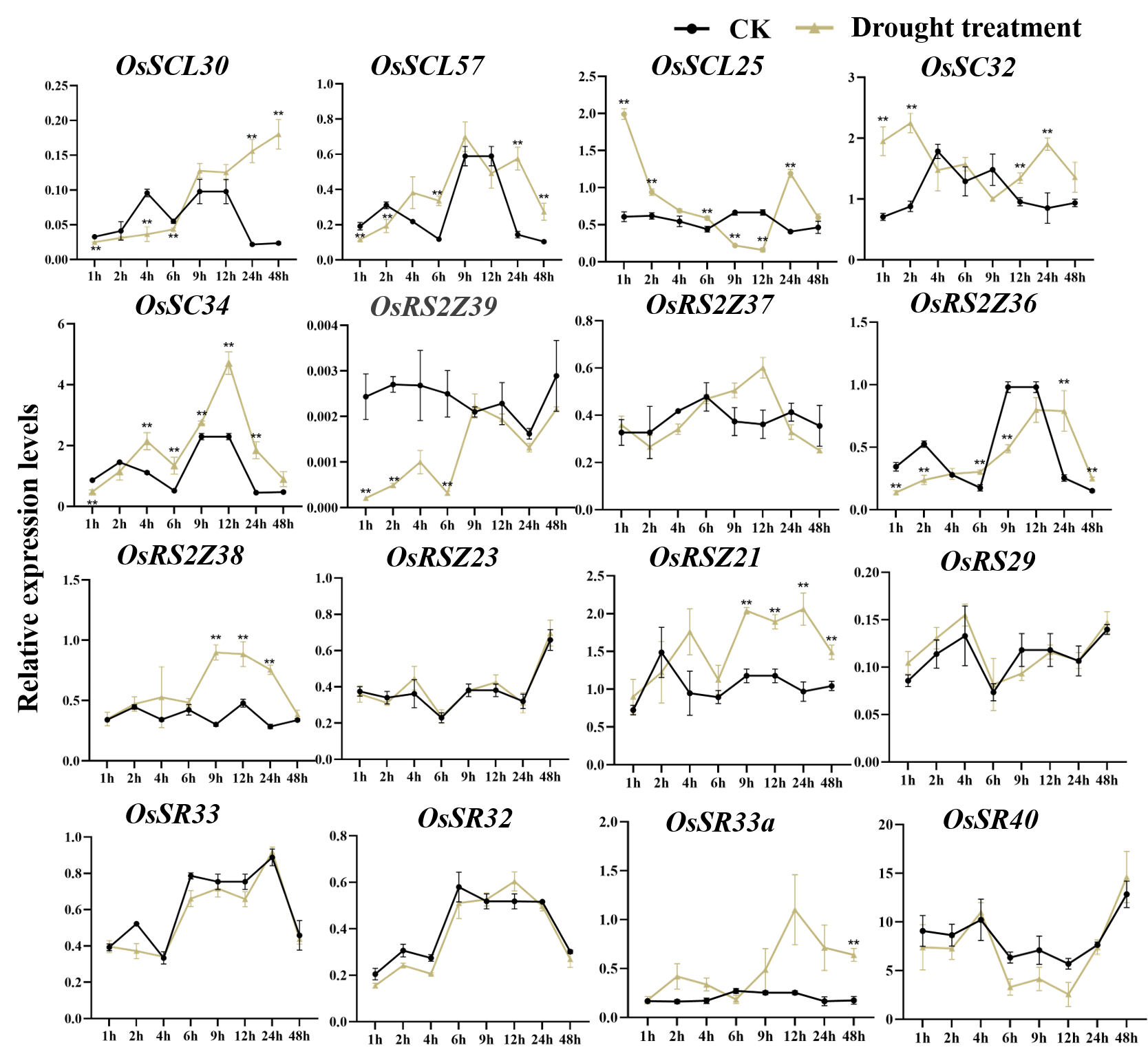

Supplement: Supplemental Information 14 — OsActin was used as control. Error bars represent mean ± SE of three biological replicates. ∗P < 0.05 and ∗∗P < 0.01 indicate significant differences com-pared with CK determined by Student’s t-test. [file peerj-11-16193-s014.png]

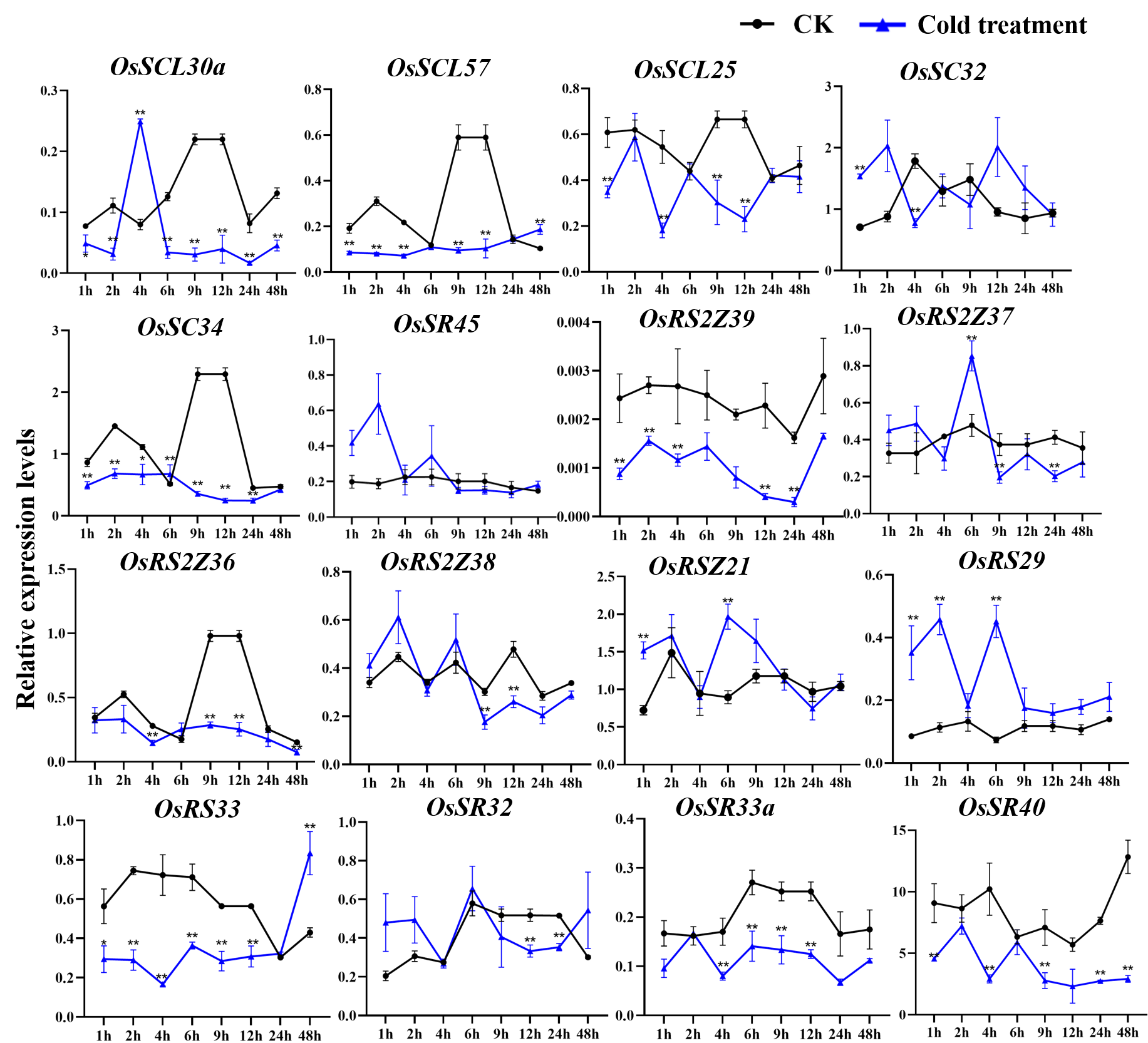

Supplement: Supplemental Information 15 — OsActin was used as control. Error bars represent mean ± SE of three biological replicates. ∗P < 0.05 and ∗∗P < 0.01 indicate significant differences com-pared with CK determined by Student’s t-test. [file peerj-11-16193-s015.png]

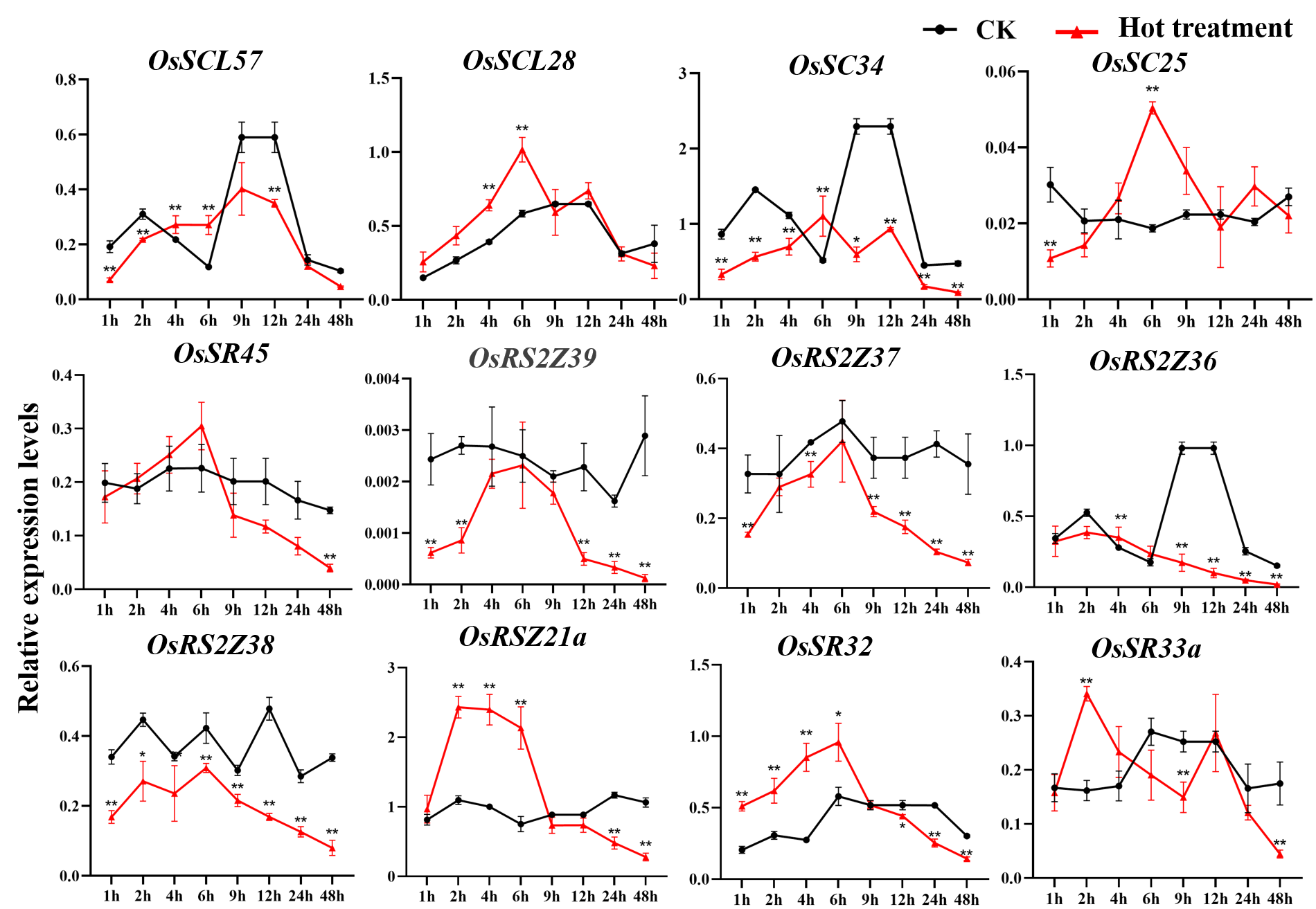

Supplement: Supplemental Information 16 — OsActin was used as control. Error bars represent mean ± SE of three biological replicates. ∗P < 0.05 and ∗∗P < 0.01 indicate significant differences com-pared with CK determined by Student’s t-test. [file peerj-11-16193-s016.png]

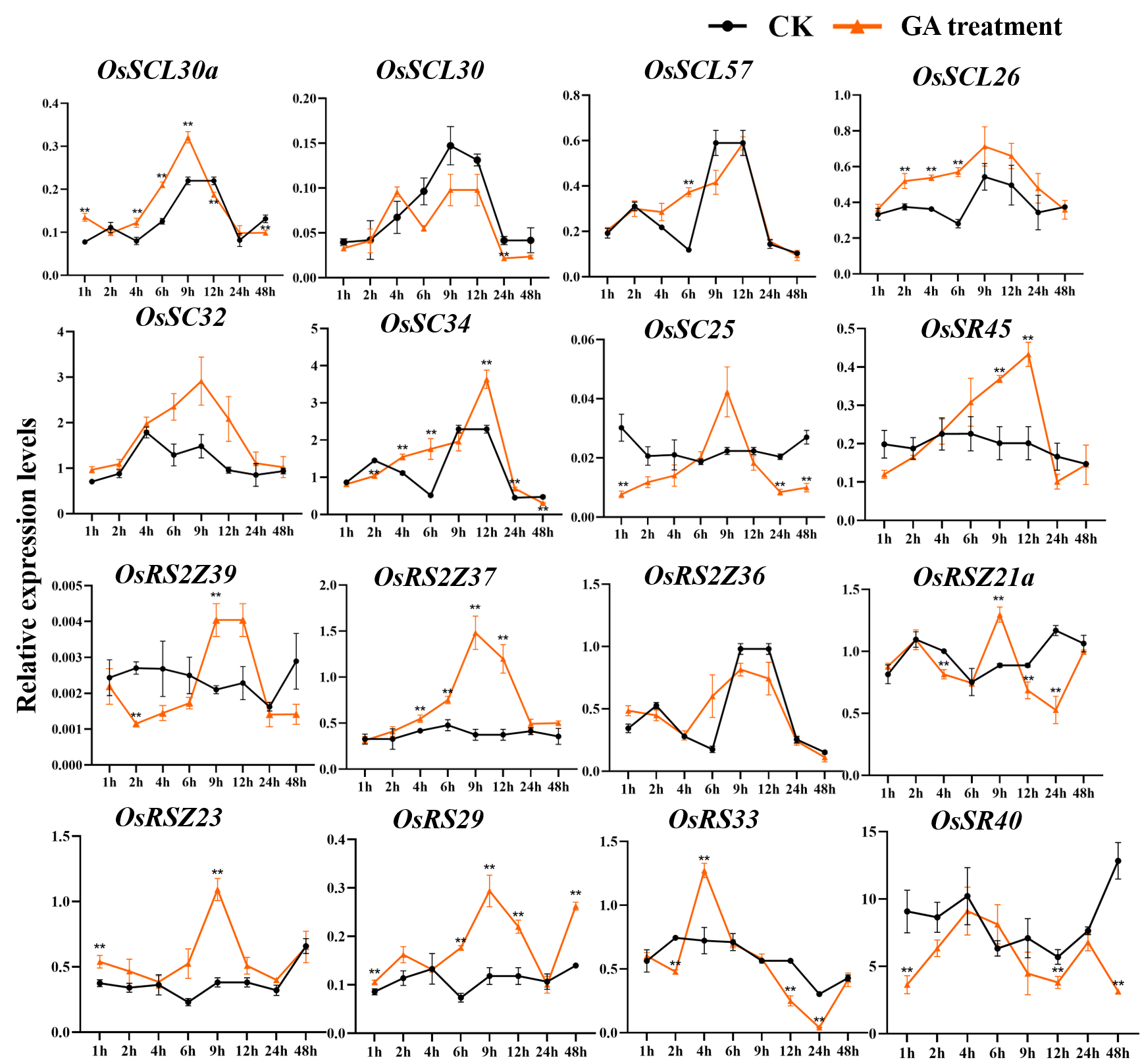

Supplement: Supplemental Information 17 — OsActin was used as control. Error bars represent mean ± SE of three biological replicates. ∗P < 0.05 and ∗∗P < 0.01 indicate significant differences com-pared with CK determined by Student’s t-test. [file peerj-11-16193-s017.png]

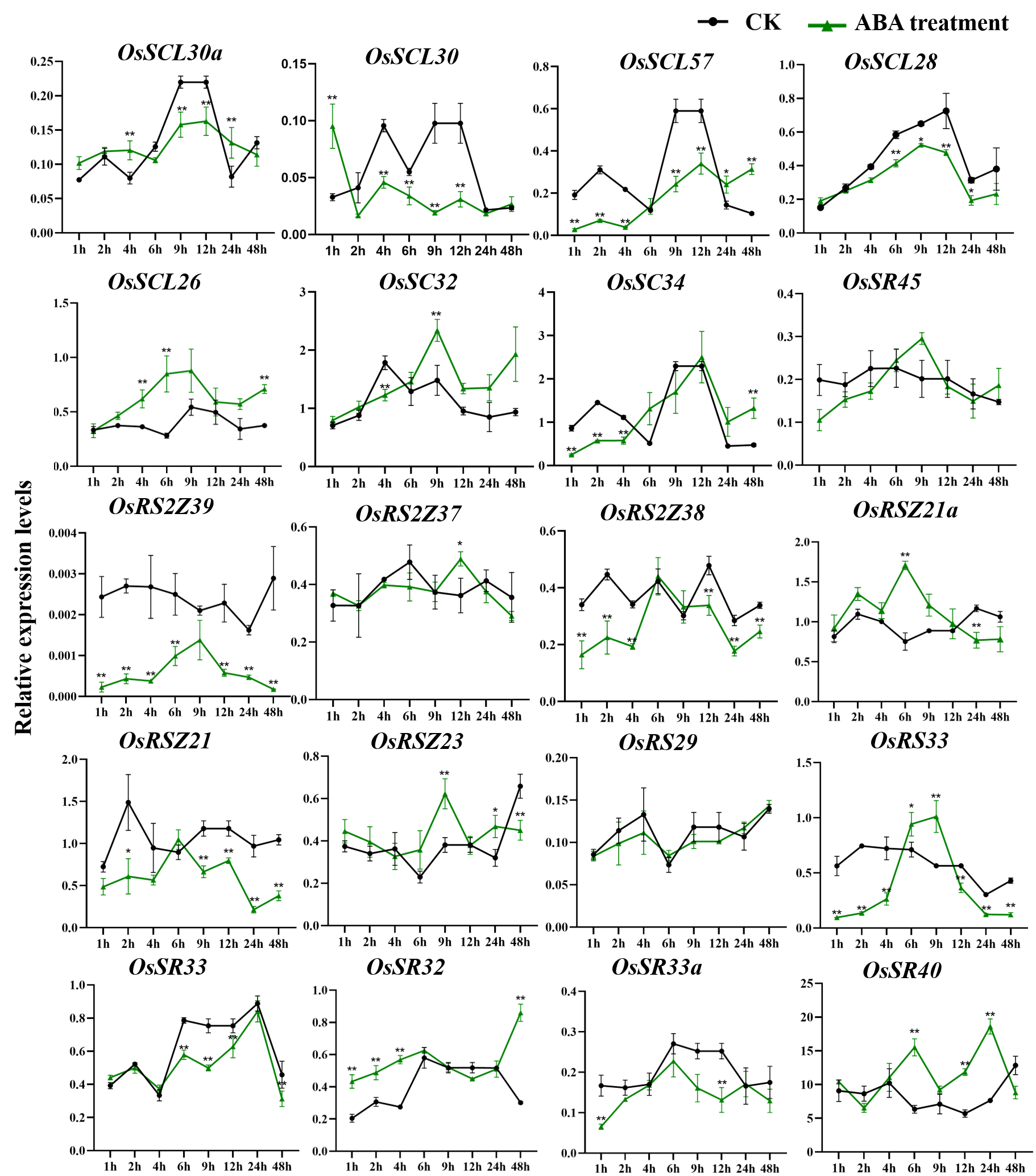

Supplement: Supplemental Information 18 — OsActin was used as control. Error bars represent mean ± SE of three biological replicates. ∗P < 0.05 and ∗∗P < 0.01 indicate significant differences com-pared with CK determined by Student’s t-test. [file peerj-11-16193-s018.png]

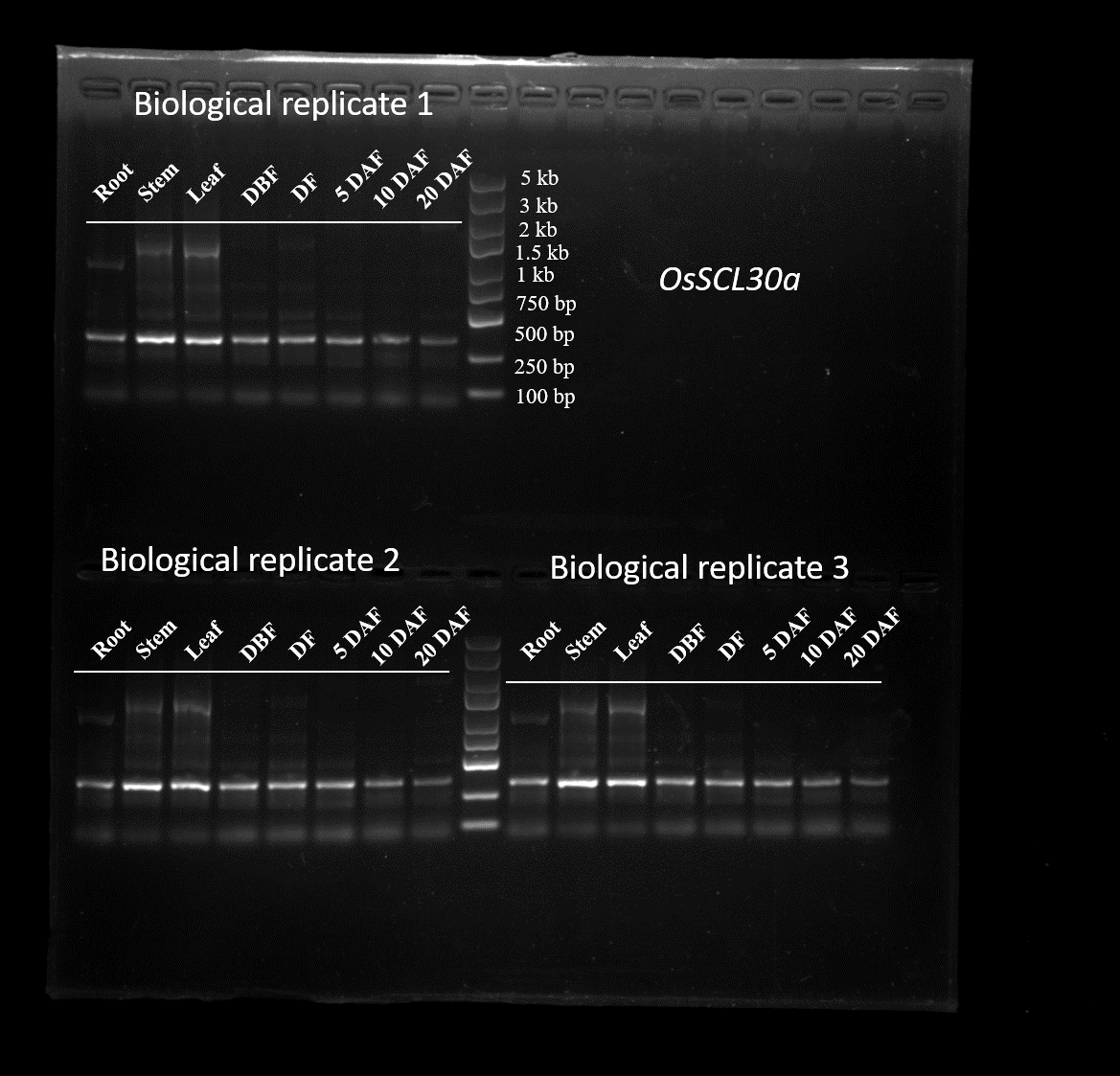

Supplement: Supplemental Information 19 [file peerj-11-16193-s019.zip › RT-PCR gels Supplemental Figure S10-21/Supplemental Figure S10.png]

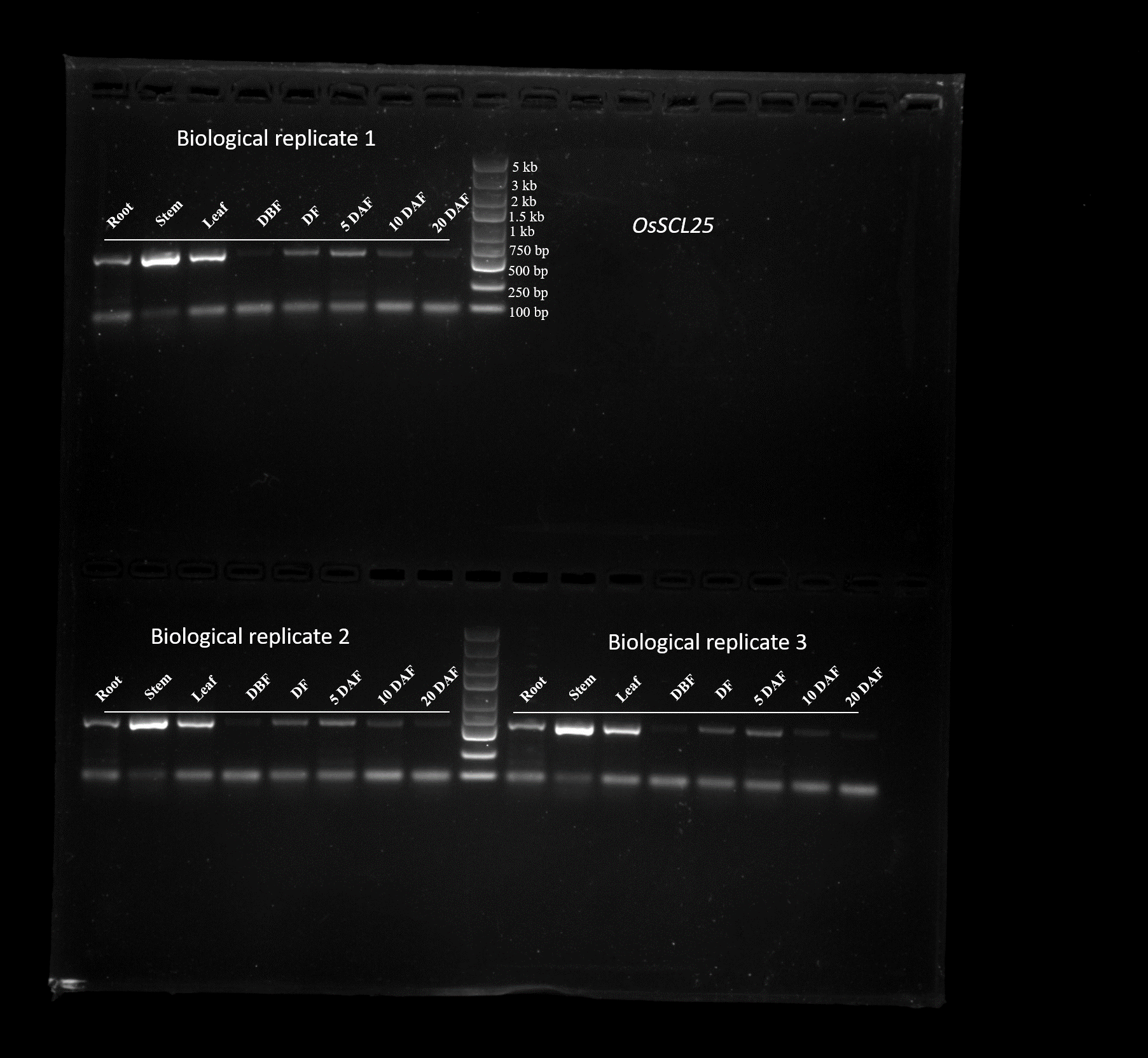

Supplement: Supplemental Information 19 [file peerj-11-16193-s019.zip › RT-PCR gels Supplemental Figure S10-21/Supplemental Figure S11.png]

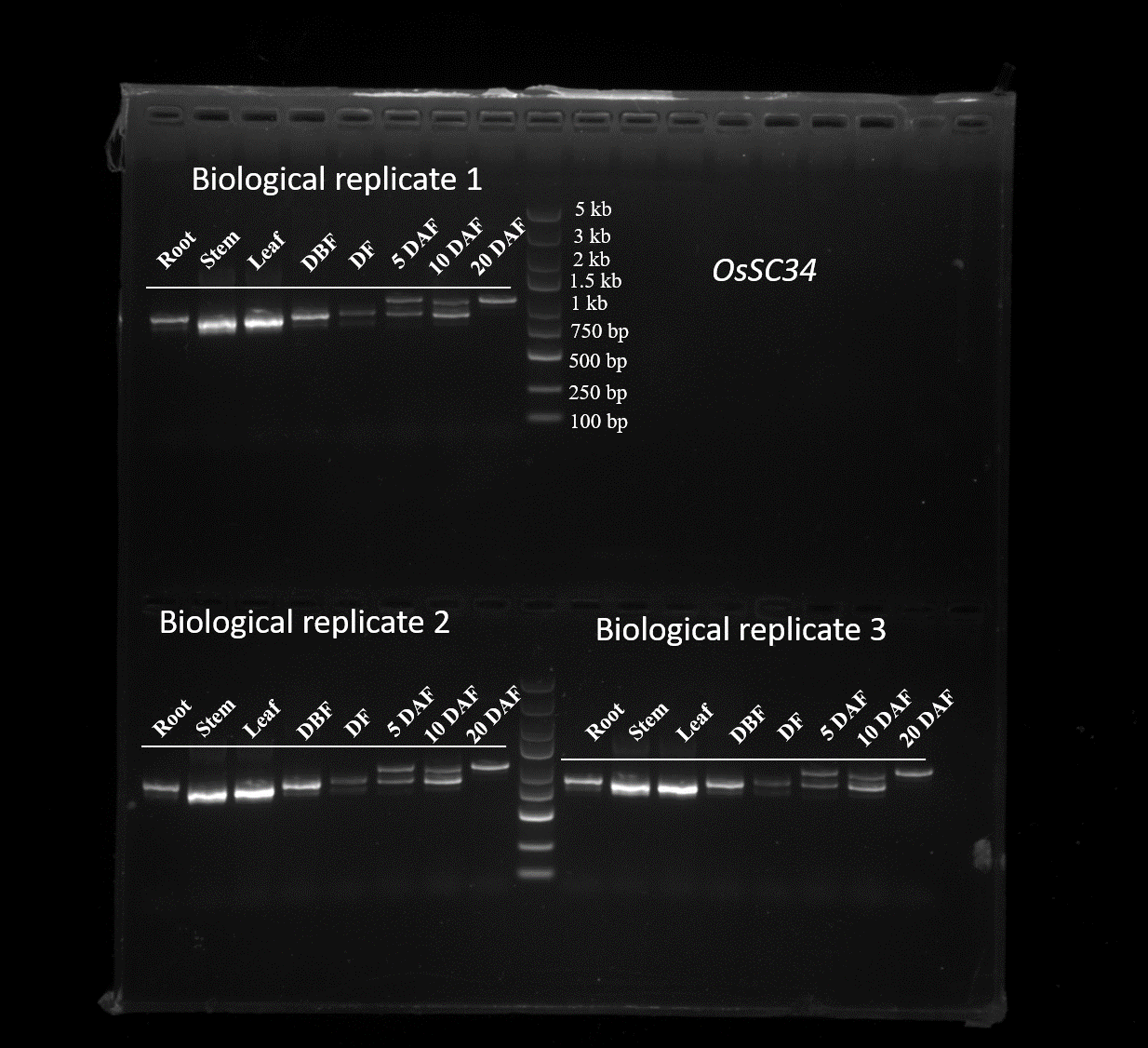

Supplement: Supplemental Information 19 [file peerj-11-16193-s019.zip › RT-PCR gels Supplemental Figure S10-21/Supplemental Figure S12.png]

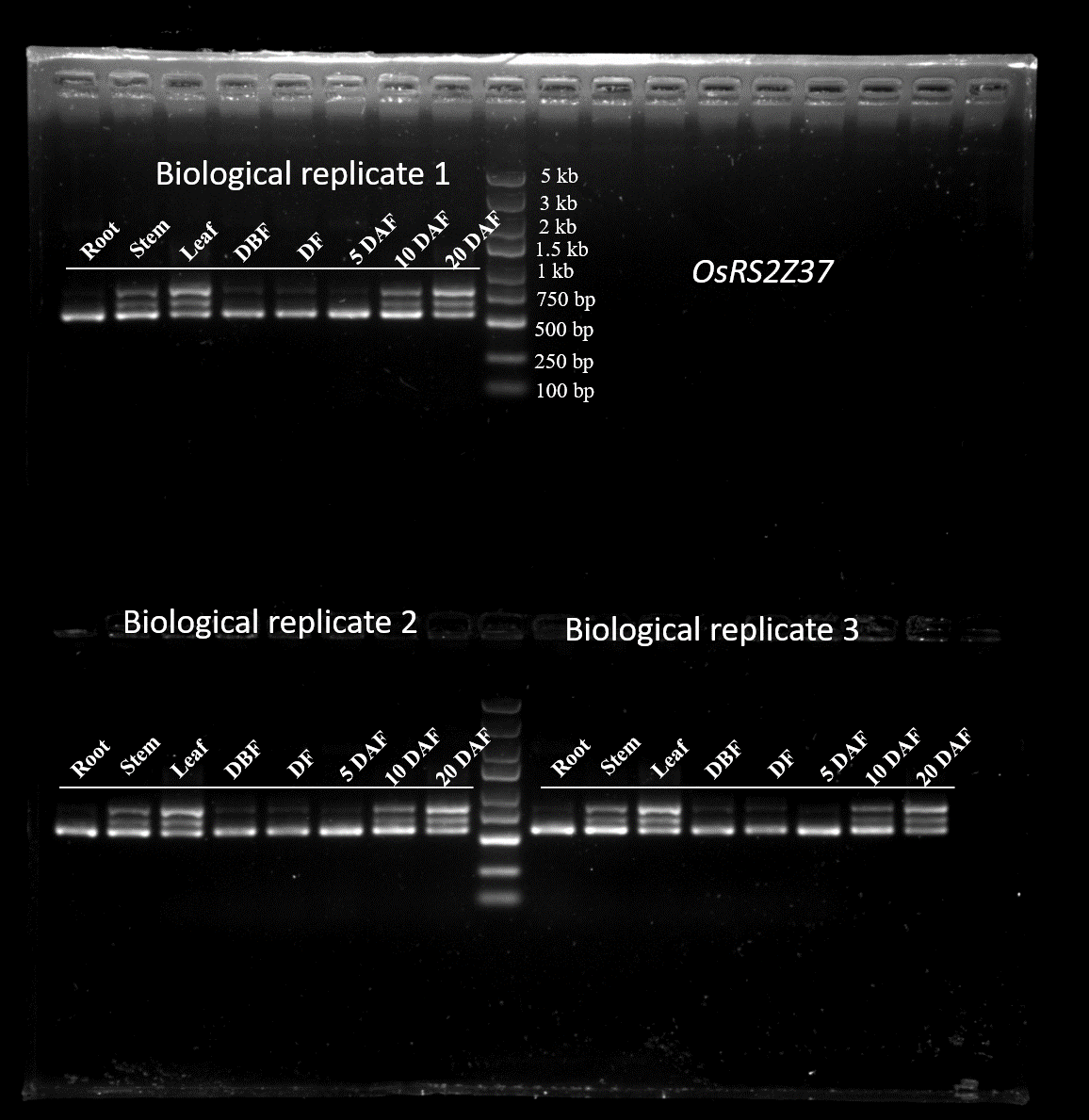

Supplement: Supplemental Information 19 [file peerj-11-16193-s019.zip › RT-PCR gels Supplemental Figure S10-21/Supplemental Figure S13.png]

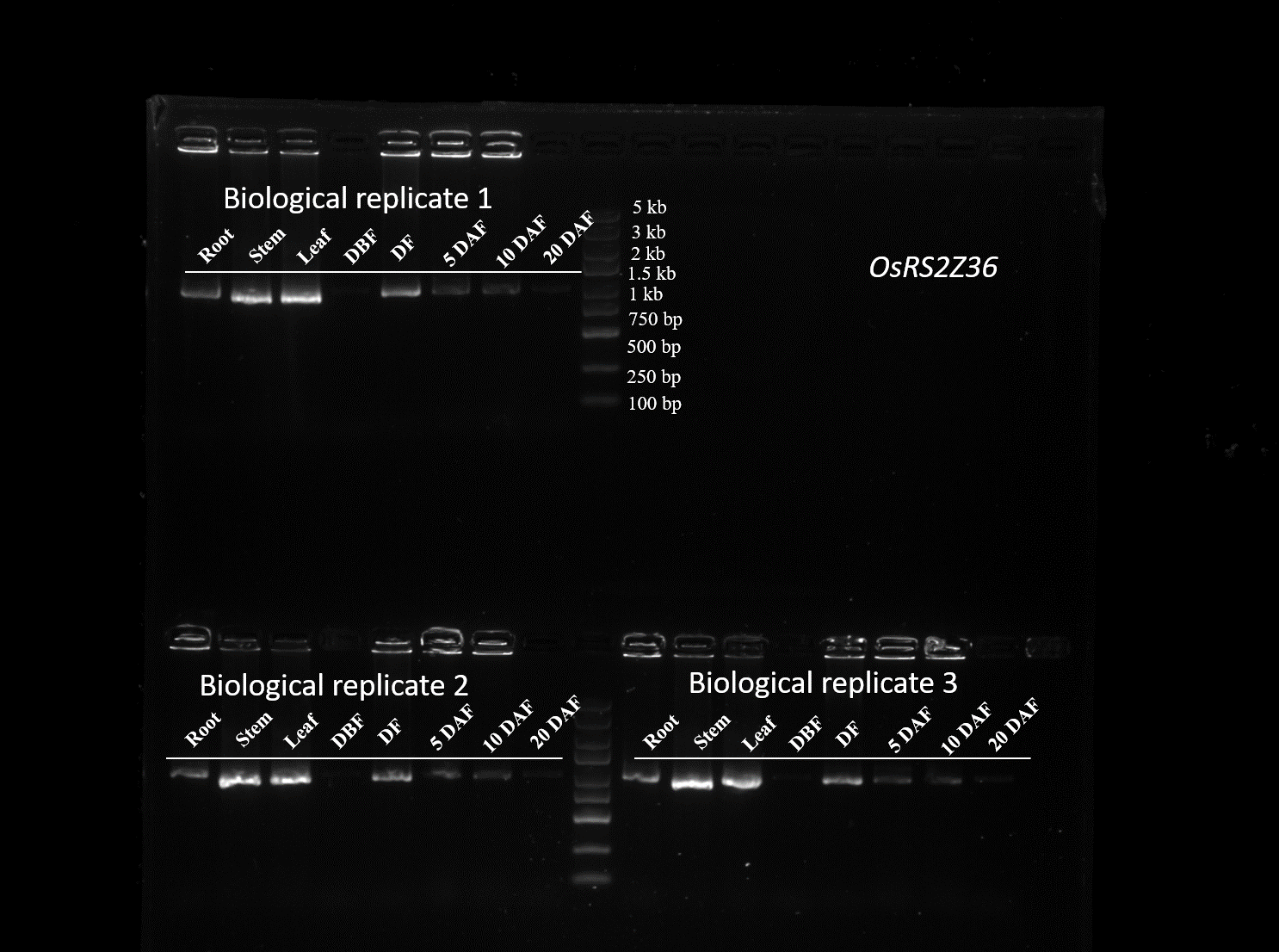

Supplement: Supplemental Information 19 [file peerj-11-16193-s019.zip › RT-PCR gels Supplemental Figure S10-21/Supplemental Figure S14.png]

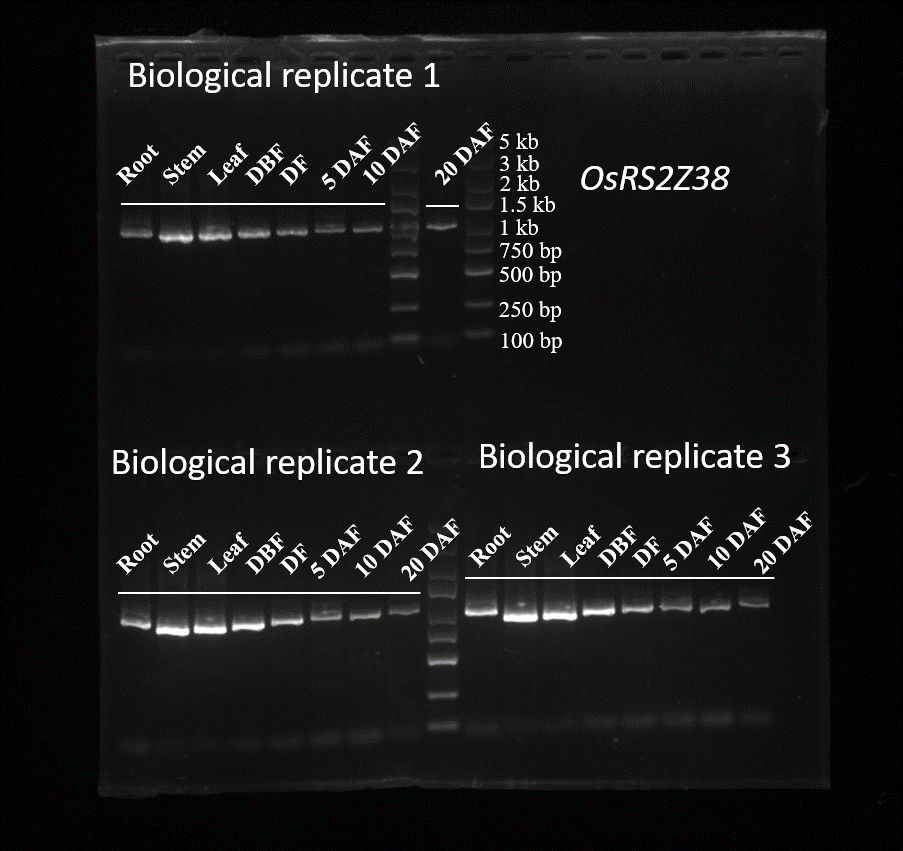

Supplement: Supplemental Information 19 [file peerj-11-16193-s019.zip › RT-PCR gels Supplemental Figure S10-21/Supplemental Figure S15.png]

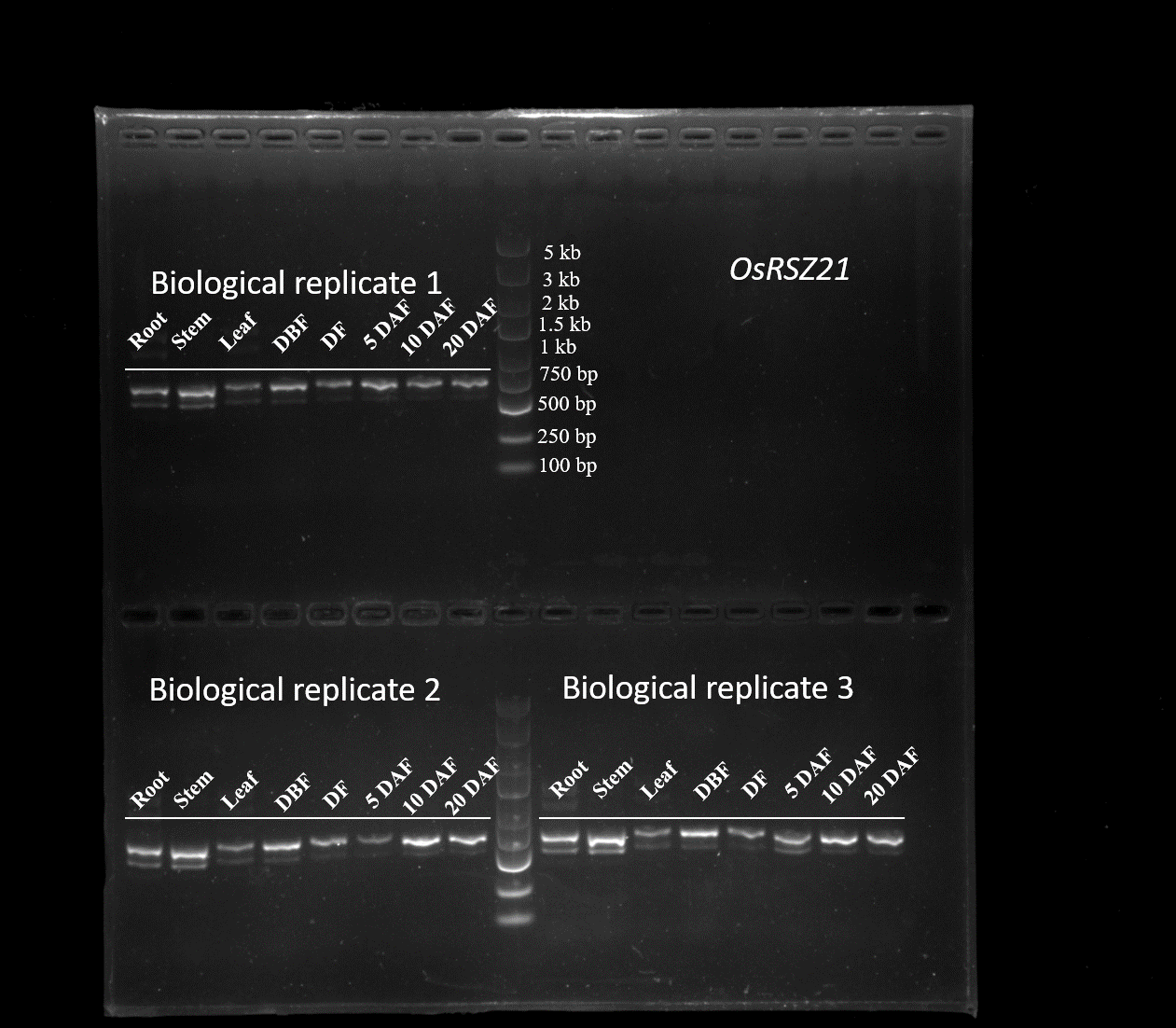

Supplement: Supplemental Information 19 [file peerj-11-16193-s019.zip › RT-PCR gels Supplemental Figure S10-21/Supplemental Figure S16.png]

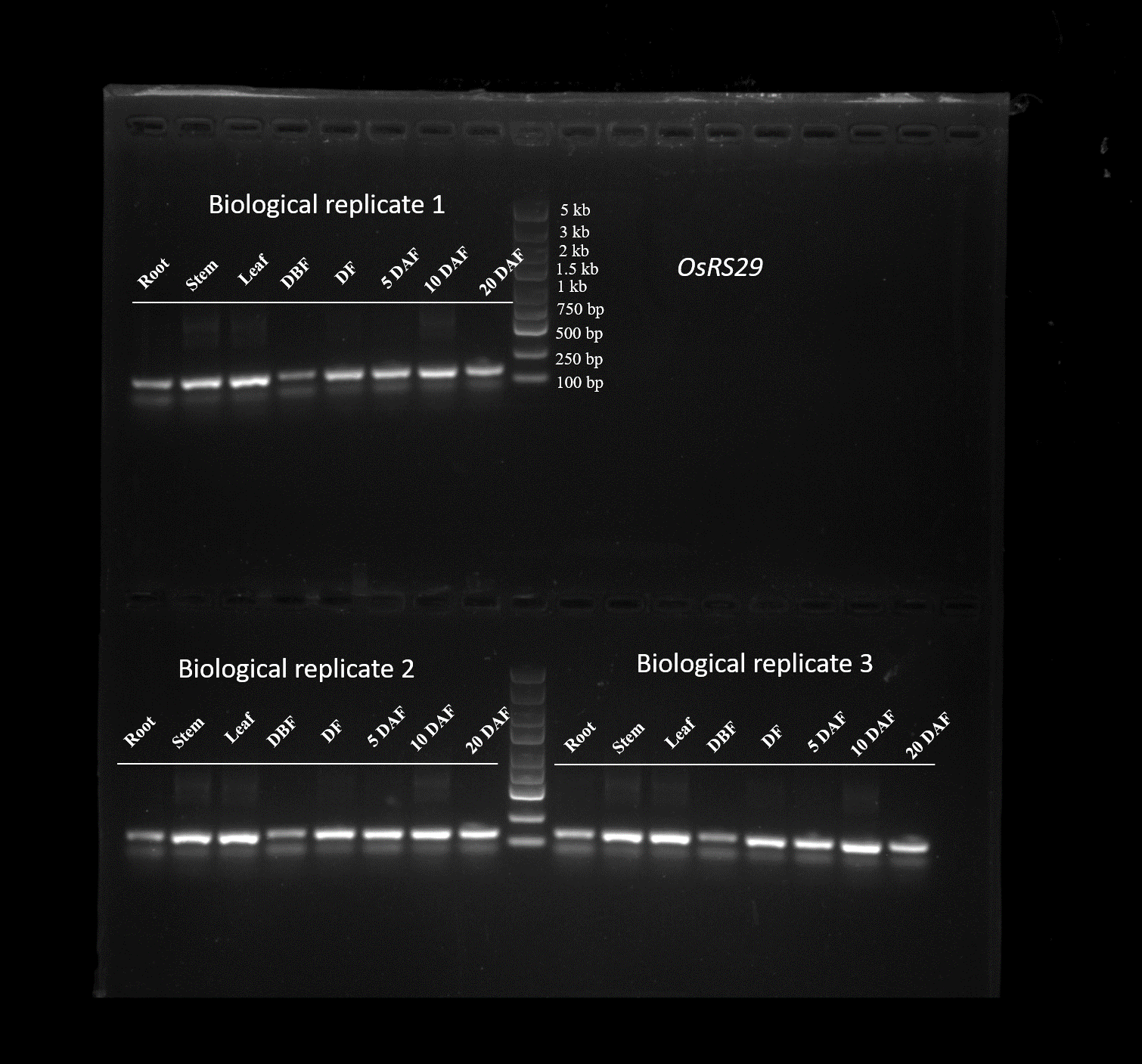

Supplement: Supplemental Information 19 [file peerj-11-16193-s019.zip › RT-PCR gels Supplemental Figure S10-21/Supplemental Figure S17.png]

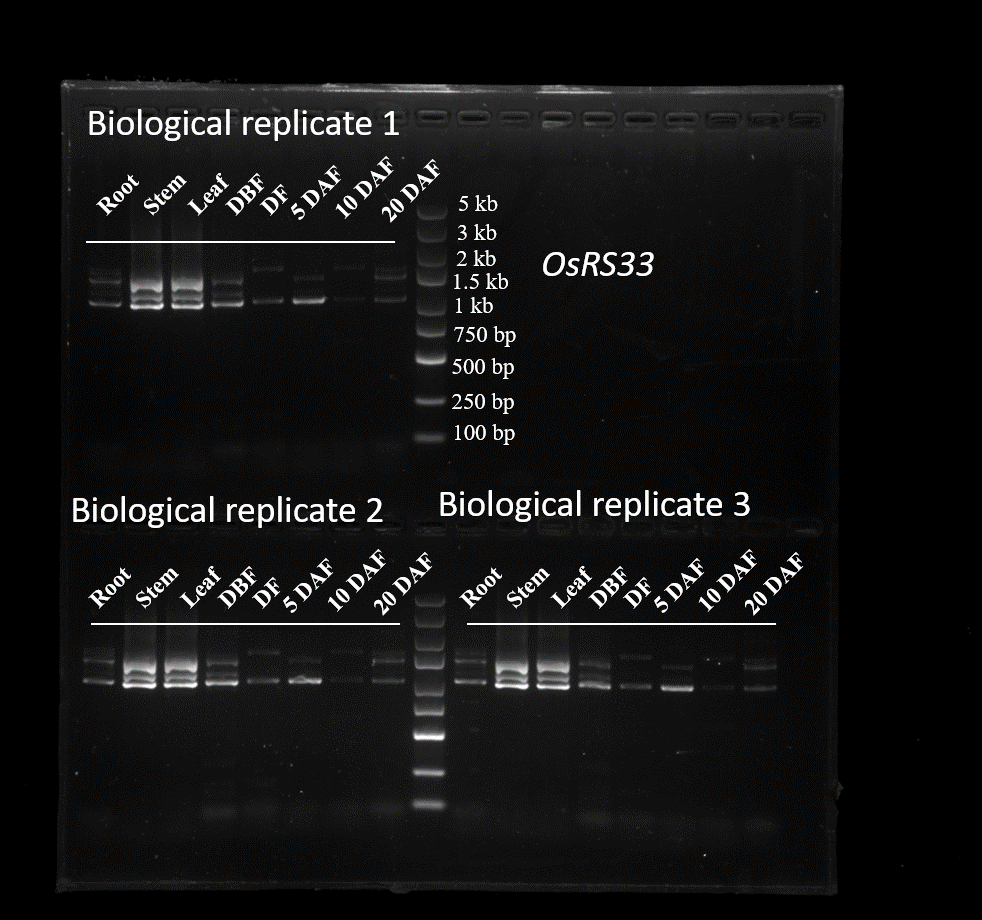

Supplement: Supplemental Information 19 [file peerj-11-16193-s019.zip › RT-PCR gels Supplemental Figure S10-21/Supplemental Figure S18.png]

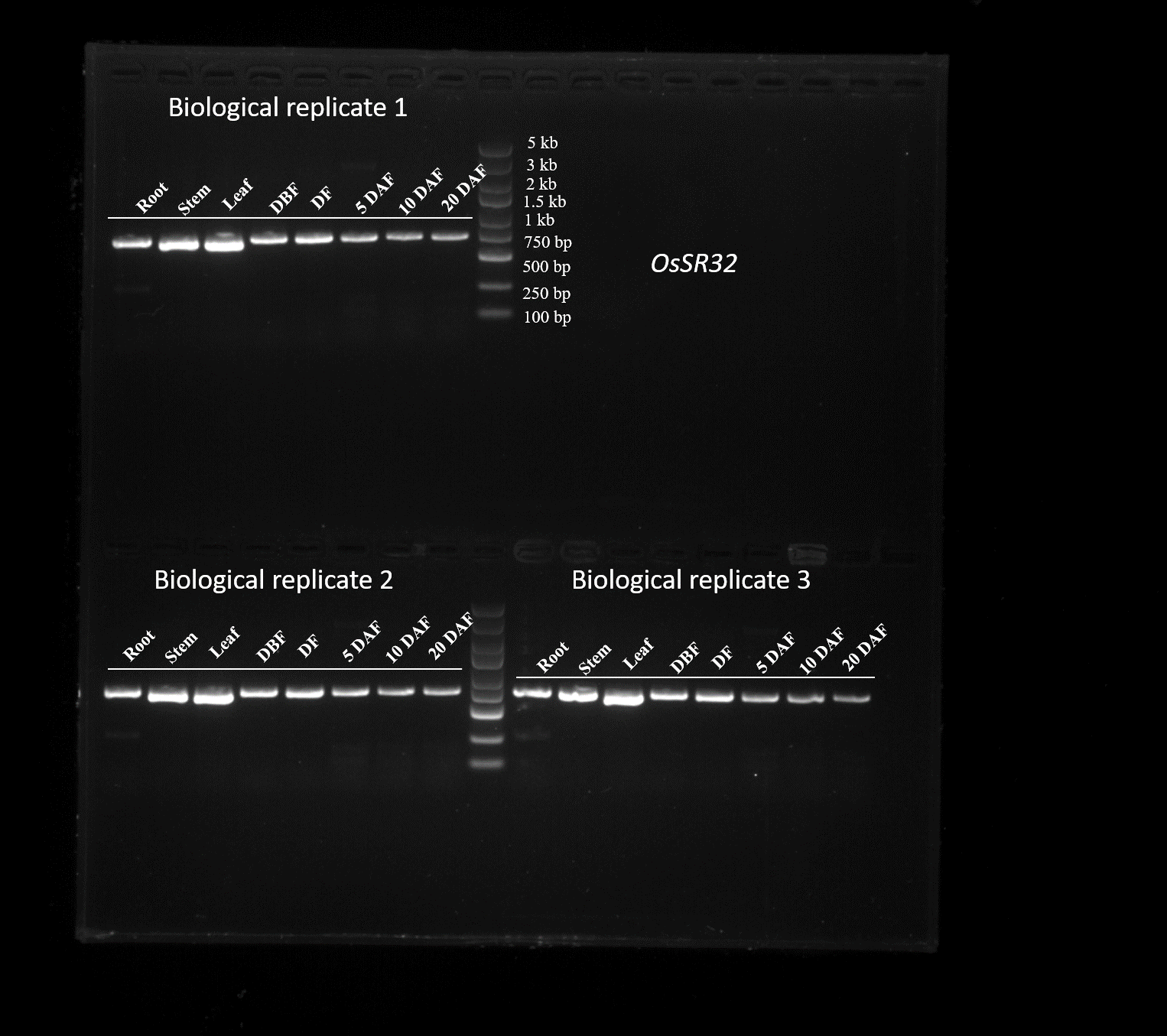

Supplement: Supplemental Information 19 [file peerj-11-16193-s019.zip › RT-PCR gels Supplemental Figure S10-21/Supplemental Figure S19.png]

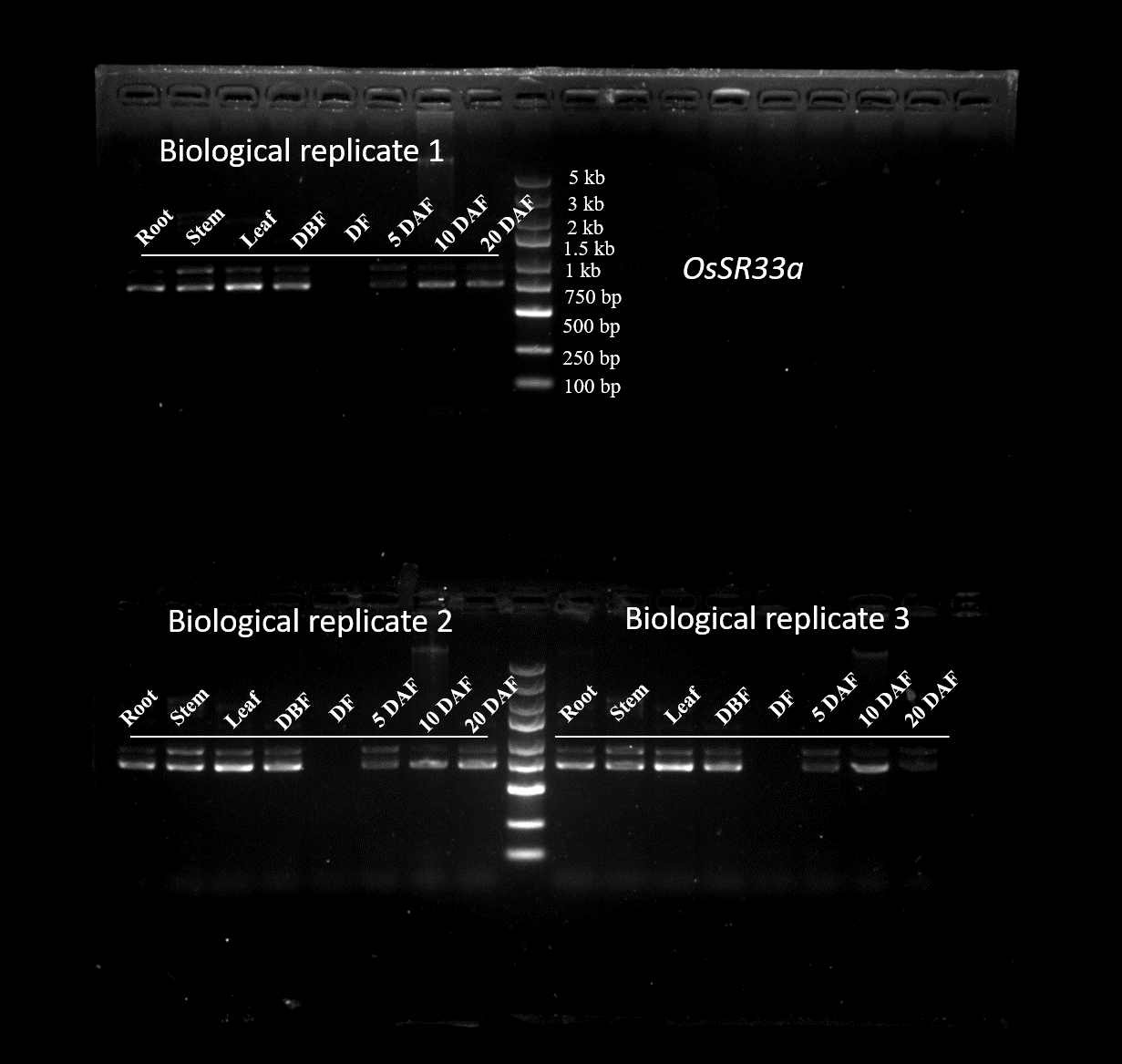

Supplement: Supplemental Information 19 [file peerj-11-16193-s019.zip › RT-PCR gels Supplemental Figure S10-21/Supplemental Figure S20.png]

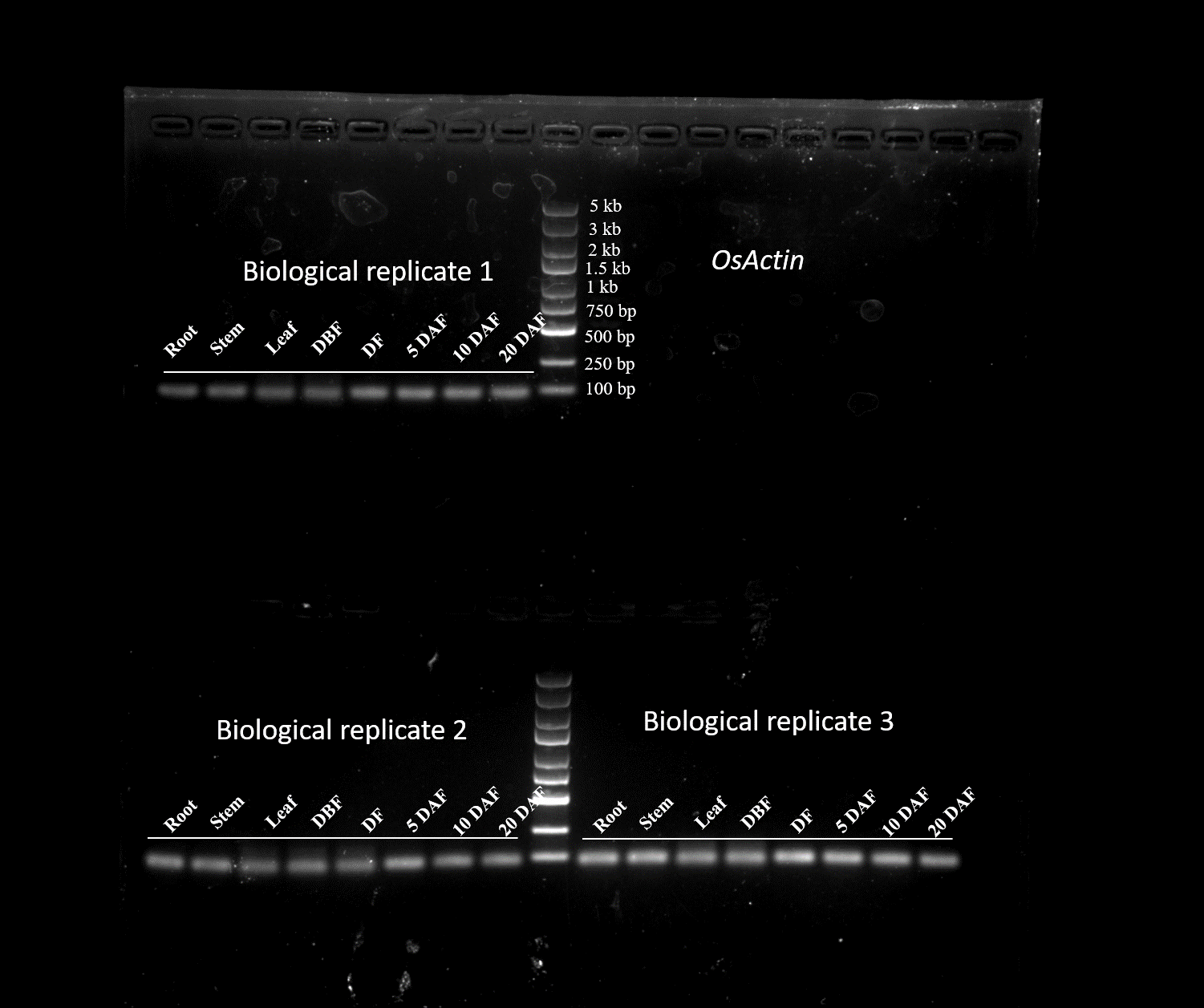

Supplement: Supplemental Information 19 [file peerj-11-16193-s019.zip › RT-PCR gels Supplemental Figure S10-21/Supplemental Figure S21.png]
